# Benefits of additional cycles of bortezomib/thalidomide/dexamethasone (VTD) induction therapy compared to four cycles of VTD for newly diagnosed multiple myeloma

**DOI:** 10.1038/s41409-019-0629-7

**Published:** 2019-07-29

**Authors:** Yoo Jin Lee, Joon Ho Moon, Sang Kyun Sohn, Seok Jin Kim, Sung-Hoon Jung, Je-Jung Lee, Jae-Cheol Jo, Ho-Jin Shin, Won Sik Lee, Ji Hyun Lee, Sung Hwa Bae, Min Kyoung Kim, Ho Sup Lee, Kihyun Kim, Chang-Ki Min, The Korean Multiple Myeloma Working Party

**Affiliations:** 1 Department of Internal Medicine, Division of Hematology and Oncology, Ulsan University Hospital, University of Ulsan College of Medicine, Ulsan, Republic of Korea; 2 Department of Hematology-Oncology, Kyungpook National University Hospital, School of Medicine, Kyungpook National University, Daegu, Republic of Korea; 3 Department of Hematology-Oncology, Samsung Medical Center, Sungkyunkwan University School of Medicine, Seoul, Republic of Korea; 4 Department of Hematology-Oncology, Chonnam National University Hwasun Hospital, Hwasun, Republic of Korea; 5 Department of Internal Medicine, Division of Hematology-Oncology, School of Medicine, Medical Research Institute, Pusan National University Hospital, Busan, Republic of Korea; 6 Department of Hematology-Oncology, Inje University Busan Paik Hospital, Busan, Republic of Korea; 7 Department of Internal Medicine, Dong-A Medical Center, Dong-A University College of Medicine, Busan, Republic of Korea; 8 Department of Hematology-Oncology, Daegu Catholic University Hospital, Catholic University of Daegu School of Medicine, Daegu, Republic of Korea; 9 Department of Hematology-Oncology, Yeungnam University Medical Center, Daegu, Republic of Korea; 10 Department of Internal Medicine, Division of hematology, Kosin University College of Medicine, Kosin University Gospel Hospital, Busan, Republic of Korea; 11 Department of Hematology, Seoul St. Mary’s Hospital, College of Medicine, The Catholic University of Korea, Seoul, Republic of Korea

**Keywords:** Myeloma, Chemotherapy

## Abstract

Bortezomib/thalidomide/dexamethasone (VTD) induction therapy followed by autologous stem cell transplantation (ASCT) is one of the standard therapies for newly diagnosed multiple myeloma (NDMM). However, the appropriate depth of response to induction therapy and timing of upfront ASCT are still debated. We investigated if two additional cycles of VTD (VTD6) improved the responses and progression-free survival (PFS) compared with four cycles of VTD (VTD4). We retrospectively reviewed outcomes of 190 NDMM patients treated with at least four cycles of VTD followed by ASCT between September 2014 and August 2017 [VTD4, *n* = 129 (67.9%); VTD6, *n* = 61 (32.1%)]. The VTD6 group had a higher pre-ASCT complete response (CR) rate than the VTD4 group (31.1% versus 10.1%, *P* < 0.001), but, the pre- and post-ASCT ≥ very good partial response (VGPR), and 2-year PFS were similar. Multivariate analysis revealed age, β_2_-microglobulin, and pre-ASCT CR as important factors for PFS. Two additional cycles of VTD prolonged PFS in patients with PR only after VTD4 [Hazard ratio (HR) = 0.29, *P* = 0.016] or those with Revised International Staging System stage I/II (HR = 0.36, *P* = 0.039). In conclusion, two additional VTD cycles may be helpful for patients with PR only after VTD4 but high risk MM needs the other treatment options.

## Introduction

Multiple myeloma (MM) still remains incurable disease despite advances in treatment with use of novel agents [1]. Initial higher quality response to new agents and their combinations is an important factor for long-term survival [2–4]. For over two decades, high-dose melphalan and autologous stem cell transplantation (HDM/ASCT) has been the standard consolidation treatment for transplant-eligible patients with newly diagnosed multiple myeloma (NDMM) to improve depth of response, progression-free survival (PFS), and likely overall survival (OS). Although overall response rate (ORR) is over 70–90% with the introduction of novel agents, it is unclear whether partial response (PR) conversion to complete response plus very good partial response (CR/VGPR) before autologous stem cell transplantation (ASCT) translate into significantly improved progression-free survival (PFS).

HDM/ASCT after 3–6 cycles of triplets induction therapy, comprising bortezomib and dexamethasone plus either an immunomodulatory drug or cyclophosphamide, is the standard treatment for patients with NDMM eligible for transplant [5–9]. Bortezomib, thalidomide, and dexamethasone (VTD) induction significantly increased the CR/VGPR rate both pre- and post-ASCT compared with bortezomib plus dexamethasone (VD), thalidomide plus dexamethasone (TD), or bortezomib, cyclophosphamide, and dexamethasone (VCD) [10–13]. However, the PETHEMA/GEM and GIMEMA studies, when only compared with TD, indicated a longer PFS for VTD [11, 12]. The IFM 2013-04 trials were unable to draw conclusions on the survival benefit in relation to higher quality of response owing to a variety of post-induction therapies [13]. Therefore, the optimal dose and schedule of VTD have not been established, although the benefits of VTD are proven.

Accordingly, in this study, whether two additional cycles of VTD after the usual 4 cycles improve pre- and post-ASCT response compared with four cycles of VTD induction therapy was evaluated. We also analysed whether PR conversion to CR/VGPR translated into significantly improved PFS. In addition, we compared the incidence and grade of adverse events in both groups.

## Materials and methods

### Data collection

The treatment outcomes in consecutive patients with NDMM between September 2014 and August 2017 from 11 hospitals of the Korean Multiple Myeloma Working Party were retrospectively reviewed. Patients below 65 years of age, with a newly diagnosed, symptomatic multiple myeloma and treated with frontline VTD followed by HDM/ASCT, were included [14]. MM was staged according to the Revised-International Staging System (R-ISS) [15]. Among 226 patients, 190 patients (84.1%), who achieved ≥PR after four cycles of VTD induction therapy, were included in this analysis. The patients were excluded in the case of progression (*n* = 11, 4.9%), unacceptable adverse events (*n* = 3, 1.3%), failure to achieve minimum threshold of 2 × 10^6^ CD34 + cells/kg (*n* = 3, 1.3%), withdrawal of consent for ASCT (*n* = 15, 6.6%), or others (*n* = 4, 1.8%). This study was approved by the Institutional Review Board of the Kyungpook National University Hospital (IRB No. 2017-11-021-004) and each participating center.

### Treatment

A frontline VTD regimen, consisting of bortezomib subcutaneous infusion (1.3 mg/m^2^ on days 1, 4, 8, and 11), thalidomide (100 mg daily, except 50 mg on days 1–14 of cycle 1), and dexamethasone (40 mg on days 1–4 and days 15–18), was administered every 4 weeks for up to 6 cycles depending on the physician’s discretion. The mobilization of autologous peripheral blood stem cells to reach a minimum threshold of 4 × 10^6^ CD34 + cells/kg was achieved by using G-CSF ± chemotherapy, and thereafter plerixafor. The administered conditioning regimen was high-dose melphalan (200 mg/m^2^ divided into two doses) followed by ASCT. A few patients (15.8%) received the maintenance therapy under institutional policies because it was not cover by national insurance.

In cases of specific predefined hematological and non- hematological toxic events, doses were modified: 1·3 mg/m² bortezomib was reduced to 1·0 mg/m², and further to 0·7 mg/m² if the first dose reduction was not effective; and 100 mg thalidomide daily was reduced to 50 mg daily. Acyclovir prophylaxis (400 mg twice daily) to prevent the reactivation of varicella zoster virus infection and acetylsalicylic acid prophylaxis (100 mg) to prevent deep vein thrombosis were recommended. Patients also received trimethoprim/sulfamethoxazole for *Pneumocystis jirovecii* prophylaxis.

### Assessments of response and adverse events

The response was assessed in accordance with the IMWG uniform response criteria [16, 17]. CR was defined as the absence of monoclonal protein in the serum and urine by immunofixation plus a normal FLC ratio of 0.26 to 1.65, on 2 consecutive assessments, along with less than 5% bone marrow plasma cells [16]. The post-transplantion response was performed at 90 days after ASCT. The bone marrow biopsy and aspirate samples were obtained at baseline, at the time to complete response was confirmed. The adverse events were assessed at every hospital visit and graded in accordance with National Cancer Institute Common Toxicity Criteria for Adverse Events (NCI-CTCAE version 4.0) [18]. Incidence rates of dose adjusted adverse events were calculated as: number of adverse events that required dose reduction/total number of patient × 100.

### Statistical analysis

The categorical data were analysed by using a chi-square test and the continuous variables were compared by using a two-sample t-test or analysis of variance (ANOVA). A logistic regression test was used to identify the factors affecting the attainment of CR. PFS was defined as the time of diagnosis to disease progression or death. The OS was measured from the time of diagnosis to death or last follow-up. The median time from diagnosis to treatment was 5 days (range, 0–33 days). PFS and OS were analysed by using the Kaplan-Meier method and log-rank test for comparison. The prognostic factors affecting PFS and OS were evaluated by using a Cox regression model. Factors with a p-value of less than 0.1 in the univariate analyses were entered in the multivariate analyses, and *p*-values of less than 0.05 were considered statistically significant. For all statistical analyses, SPSS statistical software version 20.0 (SPSS Inc.; Chicago, IL, USA) was used.

## Results

### Patient characteristics

The median age of the 190 patients was 57.0 years (range 30−64 years); 113 patients (59.5%) were men. The R-ISS was classified into 52 (27.4%), 96 (50.5%), and 40 patients (21.1%) as stage I, II, and III, respectively. Two patients (1.1%) were not classified owing to the absence of FISH result. VTD induction was administered for four cycles in 129 patients (VTD4, 67.9%) and for 5–6 cycles in 61 patients (VTD6, 32.1%) before HDM/ASCT. The patient characteristics at diagnoses did not differ between the VTD4 and VTD6 groups (Table 1).

**Table 1:** Patient demographic and clinical characteristics

*N* (%)	VTD4 (*n* = 129)	VTD6 (*n* = 61)	*P* value
Age, median (range) years	55.6 (30–63)	55.4 (40–64)	0.866
Gender			0.27
Male	73 (56.6)	40 (65.6)	
Female	56 (43.4)	21 (34.4)	
ECOG PS			
0	35 (27.1)	13 (21.3)	0.334
1	68 (52.7)	30 (49.2)	
2	26 (20.2)	18 (28.5)	
R-ISS			0.421
I	37 (28.7)	15 (24.6)	
II	61 (47.3)	35 (57.4)	
III	31 (24.0)	11 (18.0)	
Albumin, g/L, median (range)	3.75 (2.0–6.0)	3.63 (2.0–5.5)	0.379
β_2_-Microglobulin ≥ 5.5 mg/L	41 (31.8)	18 (29.5)	0.752
LDH > normal	25 (19.4)	7 (11.5)	0.215
Creatinine level, mmol/L, median (range)	1.14 (0.53–8.26)	1.27 (0.68–11.96)	0.127
Hemoglobin level, g/dL, median (range)	10.1 (6.0–16.2)	10.0 (4.0–15.0)	0.192
Calcium level, mmol/L, median (range)	9.32 (7.0–13.2)	9.22 (6.2–15.3)	0.771
Plasma cells in bone marrow ≥ 60%	52 (40.6)	23 (37.7)	0.752
FISH*			
t(4;14)	20 (18.5)	12 (26.7)	0.564
t(14;16)	5 (4.6)	6 (13.3)	0.179
t(14;20)	2 (1.6)	2 (4.1)	0.225
17p13 deletion	12 (11.1)	9 (20.0)	0.322
Amplification of 1q21	41 (40.0)	11 (24.4)	0.056

Data are presented as number (%) unless otherwise indicated. VTD4 indicates four cycles of bortezomib, thalidomide, and dexamethasone; and VTD6 indicates six cycles of bortezomib, thalidomide, and dexamethasone. *ECOG PS* Eastern Cooperative Oncology Group performance status, *R-ISS* revised international staging system, *LDH* lactate dehydrogenase, *FISH* fluorescent in situ hybridization

### Response rates after additional cycles of VTD

The pre- and post-ASCT responses are shown in Table 2. The CR/VGPR rate after four cycles of VTD induction was significantly lower in the VTD6 group (*n* = 28/61, 45.9% vs *n* = 103/129, 79.8%, *P* < 0.001). After two additional cycles of VTD, the CR/VGPR rates of pre- and post-ASCT were similar between two groups. Furthermore, the pre-ASCT CR rate was significantly higher in the VTD6 than in the VTD4 group (31.1 vs 9.3%, *P* < 0.001); however, the post-ASCT CR rate was not different between the two groups (72.1 vs 62.3%, *P* = 0.183).

**Table 2:** Comparisons of Response Rates between VTD4 and VTD6

	VTD4	VTD6		*P*-value^a^
	At 4 cycles	At 4 cycles	At 6 cycles	
Pre-transplant response				
≥Complete remission	13 (10.1)	6 (9.8)	19 (31.1)	<0.001
≥Very good partial response	103 (79.8)	28 (45.9)	41 (67.2)	0.077
≥Partial response	129 (100)	61 (100)	61 (100)	–
Post-transplant response at 3 months				
≥Complete remission	93 (72.1)	–	38 (62.3)	0.183
≥Very good partial response	111 (86.0)	–	51 (83.6)	0.666
≥Partial response	126 (97.7)	–	54 (98.4)	0.758

VTD4 indicates four cycles of bortezomib, thalidomide, and dexamethasone; VTD6 indicates six cycles of bortezomib, thalidomide, and dexamethasone

^a^Comparison of pre-ASCT response rates

The probability of achieving a higher quality of response depending on the cumulative treatment of VTD induction and HDM/ASCT is shown in Figure S1. In the VTD6 group, 20 patients (32.7%) obtained a deeper response after two additional cycles of VTD (Table S1).

### Stem cell mobilisation and transplantation

The median time between day 1 of the final VTD cycle and stem cell infusion was 10.4 weeks (95% confidence interval [CI]: 3.0–27.4 weeks) and 10.1 weeks (95% CI: 2.3–22.4 weeks) in the VTD4 and VTD6 groups, respectively (*P* = 0.944). There was no significant difference in the mobilisation time between the two groups. However, the median number of CD34 + cells was 4.01 × 10^6^ /kg (2.0–62.0 × 10^6^/kg) in the VTD4 and 8.00 × 10^6^/kg (2.0–52.0 × 10^6^/kg) in the VTD6 group (*P* = 0.001). The number of patients using chemotherapy or plerixafor was significantly higher in the VTD6 group (38.3% vs 65.6%, *P* = 0.001). There was no difference in neutrophil or platelet engraftment between the groups. No toxic death was recorded during the HDM/ASCT procedure.

### Adverse events with additional cycles of VTD

Newly developed or aggravated adverse events in every two cycles of VTD are shown in Table 3. The incidence of any grade of gastrointestinal symptoms in cycle 1 and 2 was higher in VTD 4 than VTD6 (38.8% vs 18.0%, *P* = 0.004). The incidence of any grade of peripheral neuropathy (PN) in cycles 1 through 4 was also higher in the VTD4 group than in the VTD6 group (Table 3). However, the incidence of grade ≥ 2 PN in cycles 1 through 4 was not significantly different in the two groups. BM suppression was similar in both groups. Moreover, the incidence of dose adjusted adverse events was similar in the VTD4 and VTD6 groups (Figure S3). However, the intensity of required dose reduction following exposure times was greater in the VTD4 group than the VTD6 group. Nevertheless, 34.4% of the VTD6 group, who were relatively tolerable to PN, experienced newly developed or exacerbated PN in cycle 5 and 6.

**Table 3:** Adverse events accompanied by VTD cycles

	1~2 cycles			3~4 cycles			5~6 cycles
*N* (%)	VTD4	VTD6	*P*	VTD4	VTD6	*P*	VTD6
**Any grade**							
Gastrointestinal	50 (38.8)	11 (18.0)	0.004	12 (9.3)	4 (6.6)	0.591	3 (4.9)
PN^a^	59 (45.7)	18 (29.5)	0.026	82 (63.6)	28 (45.9)	0.021	30 (49.2)
BM suppression	20 (16.5)	11 (18.0)	0.66	7 (5.4)	4 (6.6)	0.748	2 (3.3)
Infection	4	5	–	4	2	–	
Herpes Zoster	0	1	–	3	1	–	1
Thrombosis	1	0	–	0	0	–	0
Fatigue	16	6	–	1	0	–	1
Others^b^	2	2		0	2		
**≥ Grade 3**							
Gastrointestinal	8 (6.2)	2 (3.3)	0.506	2 (1.6)	0	1	0
PN	9 (7.0)	6 (9.8)	0.052	27 (20.9)	13 (21.3)	0.95	11 (18.0)
BM suppression	6 (4.7)	1 (1.6)	0.433	4 (3.1)	1 (1.6)	0.673	1 (1.6)

^a^Peripheral neuropathy ≥ Grade 2

^b^Others comprised: skin rash, edema, dizziness

VTD4 indicates four cycles of bortezomib, thalidomide, and dexamethasone; VTD6 indicates six cycles of bortezomib, thalidomide, and dexamethasone. *PN* peripheral neuropathy, *BM* bone marrow

### Survival outcomes in overall patients

The median follow-up duration was 23.4 months (range, 7.0–47.2 months). The median follow-up duration was 23.6 months (7.0–47.2 months) in VTD4 and 21.3 months (9.0–34.0 months) in VTD6.

**Fig. 1:**
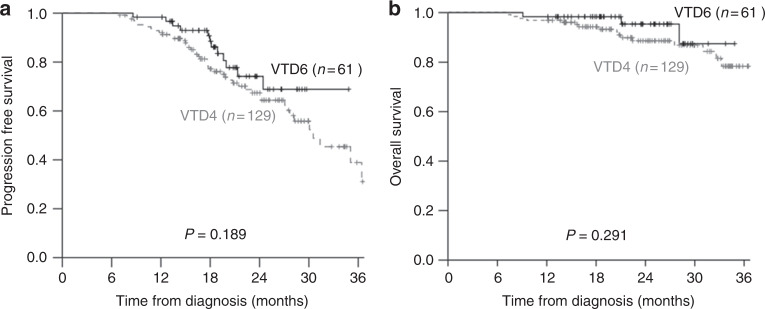
Survival rates according to the two additional cycles of VTD. **a** Progression-free survival (PFS): The 2-year PFS rate was 64.4 ± 5.0% and 68.9 ± 8.3% for the VTD4 and VTD6 groups, respectively (*P* = 0.189). **b** Overall survival (OS): The 2-year OS rate was 88.6 ± 3.2% and 95.4 ± 3.4 % for the VTD4 and VTD6 groups, respectively (*P* = 0.291)

The median PFS was 30.5 months (range, 25.0–36.0 months) for the VTD4 group and was not reached for the VTD6 group (*P* = 0.370). The median OS has not been reached for either group. The 2-year PFS rate was 64.4% ± 5.0% and 68.9% ± 8.3% for the VTD4 and VTD6 groups, respectively (*P* = 0.189, Fig. 1a). The 2-year OS rate was 88.6% ± 3.2% and 95.4% ± 3.4% for the VTD4 and VTD6 groups, respectively (*P* = 0.291, Fig. 1b). Multivariate analysis revealed that age, β_2_-microglobulin, and pre-ASCT CR were important prognostic factors for PFS (Table 4). Lactate dehydrogenase and post-ASCT CR were associated significantly with OS by multivariate analysis (Table 4).

**Table 4:** Multivariate analysis of factors affecting on survival outcomes

	Progression-free survival		Overall survival	
	HR (95% CI)	*P*-value	HR (95% CI)	*P*-value
Age	0.95 (0.92–0.99)	0.012	–	–
LDH > normal	–		3.01 (.38–6.58)	<0.001
β2 microglobulin ≥ 5.5 mg/L	2.91 (1.68–5.02)	<0.001	–	–
Pre-ASCT			–	–
CR	1			
VGPR	3.14 (1.20–8.21)	0.05	–	–
PR	5.96 (1.93–18.39)	0.009	–	–
Post-ASCT at 3 months				
CR	1		1	
VGPR	1.24 (0.68–2.25)	0.557	4.21 (1.71–10.37)	0.009
PR	1.97 (0.86–4.54)	0.179	2.15 (0.56–8.24)	0.35
PD	53.2 (15.8–178.9)	<0.001	23.7 (7.13–78.75)	<0.001

*HR* hazard ratio, *CI* confidence interval, *LDH* lactate dehydrogenase, *CR* complete response, *VGPR* very good partial response, *PR* partial response, *PD* progressive disease

### Advantage of additional cycles of VTD in patients with PR

**Fig. 2:**
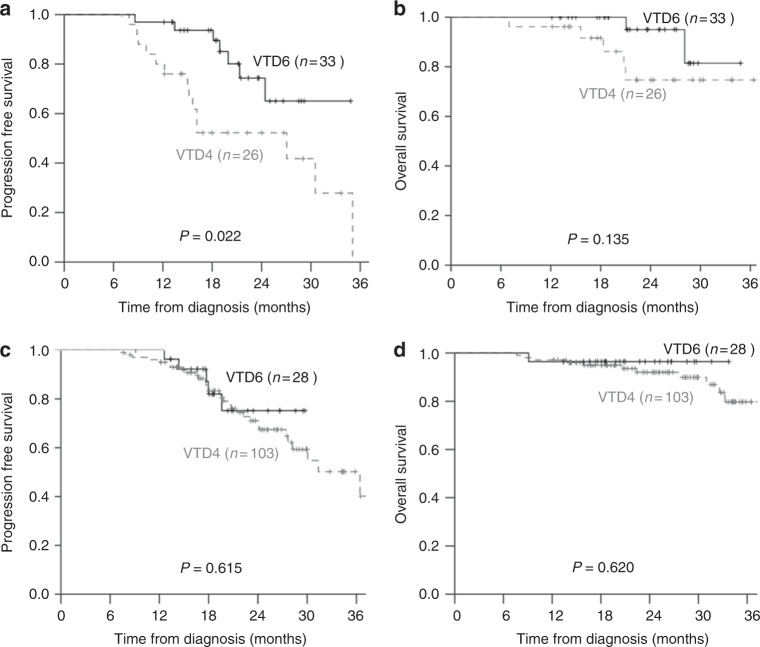
Survival rates in the VTD4 and VTD6 groups according to the response after four cycles of VTD. Patients with partial response only after four cycles of VTD showed superior (**a**) 2-year Progression-free survival (PFS) in the VTD6 groups (74.4 vs 52.2%, *P* = 0.022). **b** The 2-year overall survival (OS) rate in these patients was not different. Patients who already achieved CR/VGPR after four cycles of VTD showed similar survival rates. **c** The 2-year PFS was 67.4 ± 5.6% and 75.2 ± 10.0% in VTD4 and VTD6 groups, respectively (*P* = 0.615). **d** The 2-year OS was 92.0 ± 3.0% and 96.4 ± 3.5% in VTD4 and VTD6 groups, respectively (*P* = 0.620)

The number of patients with PR only after four cycles of VTD was higher in VTD6 than VTD4 (54.1 vs 20.2%, *P* < 0.001). There were no significant differences in FISH abnormalities or R-ISS between the two groups. To assess the benefit of two additional cycles of VTD, PFS and OS were analysed for the 59 patients who achieved PR only after four cycles of VTD. The VTD6 group had significantly higher PFS (median PFS; not reached [NR] vs 27.1 months (12.9–41.2 months, *P* = 0.022, Fig. 2a). But OS was not different between two groups (*P* = 0.135, Fig. 2b). In the multivariate analysis of this subgroup, young age, non-high risk of R-ISS stage I/II, and two addition cycles of VTD were independently favorable prognostic factors for PFS (Table S2). On the one hand, the patients who had already achieved CR/VGPR at four cycles of VTD did not show the survival benefit with two additional cycles of VTD (Fig. 2c, d).

As expected, the patients who obtained a deeper response with two additional cycles of VTD indicated prolonged PFS compared with the patients who did not achieve a deeper response (2-year PFS; 89.8% ± 6.9% vs 62.2% ± 10.7%, *P* = 0.050).

### Advantage of additional cycles of VTD in non-high risk patients

The median PFS was not reached, 31.4 months (25.9–36.8 months), and 21.5 months (16.2–26.7 months) for the stage I, II, and III by R-ISS, respectively (*P* < 0.001, Figure S2A). For the 148 patients with R-ISS stage I/II, the 29 patients (58.0%) achieved the higher quality of response although only two patients (*n* = 2/11, 18.2%) with R-ISS stage III experienced an improved quality of response from two additional cycles of VTD (Table S1).

**Fig. 3:**
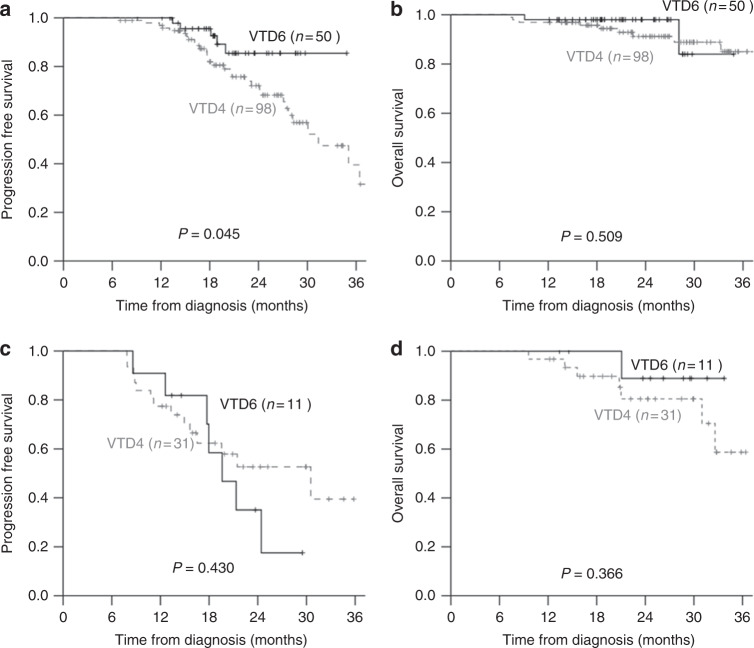
Survival benefit from two additional cycles of VTD by Revised International Staging System (R-ISS). Patients with R-ISS stage I/II showed superior (**a**) The 2-year Progression-free survival (PFS) in the VTD6 group (67.8 ± 6.3% vs 90.2 ± 6.9%, *P* = 0.045). **b** The 2-year overall survival (OS) rate in these patients was not different. Patients with R-ISS stage III showed similar 2-year PFS (**c**) and the 2-year OS (**d**)

The patients with R-ISS stage I/II showed prolonged PFS when they received additional two cycles of VTD (median PFS; NR vs 31.4 months, *P* = 0.045, Fig. 3a), although the patients with R-ISS stage III did not obtain the benefit of PFS (Fig. 3c, *P* = 0.43). In the multivariate analysis of this subgroup, two additional cycles of VTD and the absence of t(4;14) in FISH were independently important factors for PFS (Table S3). There was no difference in OS rate for between two groups regardless of R-ISS.

## Discussion

HDM/ASCT has been adopted as a standard care for NDMM, however, the appropriate depth of response and timing for pre-transplantation therapy for upfront ASCT is still debated. The current study evaluated whether two additional cycles of VTD improved the pre- and post-ASCT responses as well as PFS compared with the four cycles of VTD induction therapy. Our study demonstrated that two additional cycles of VTD resulted in a higher pre-ASCT CR rate than that in the VTD4 group. However, the pre- and post-ASCT CR plus VGPR rates and post-ASCT CR rate were not different in the two groups. The prolonged PFS from two additional cycles of VTD was found in the patients with PR only after four cycles of VTD or the patients with R-ISS stage I/II at diagnosis although new onset or aggravated PN during additional treatment occurred in 34.4% of the patients.

In the current study, two additional cycles of VTD resulted in a higher pre-ASCT CR rate than in the VTD4 group. Several studies have already shown a correlation between the quality of response and the long-term survival outcomes [3, 4]. The current study also consistently showed that pre-ASCT CR was an independently prognostic factor for PFS (Table 4). In contrast with previous studies in which the proportion of patients exposed to bortezomib was less than 10%, the current study in which all patients were exposed to bortezomib compared the VTD6 group with the VTD4 group. There was no difference in 2-year PFS and OS between two groups (Figs. 2a, b). Although the VTD6 group included more patients with poor prognosis, such as less favorable responses and high risk features in FISH, similar survival outcomes suggest that two additional cycles of VTD might allow the induction of a deeper response and improve PFS. As expected, PR conversion to CR/VGPR from two additional cycles of VTD seem to decrease the risk of disease progression when the patients attained only PR after four cycles of VTD induction therapy (HR = 0.29, *P* *=* 0.016, Table S2).

In the current study, the patients who failed to achieve at least PR after four cycles of VTD or did not proceed to ASCT were not included to investigate the benefit of two additional cycles of VTD for transplant eligible NDMM. In the real world, the exclusion rate was higher than the previous study (15.9% vs 7.0–10.0%) [11–13]. Therefore, CR rates of selected patients represented higher than expected. The previous studies also showed a various pre-transplant CR rate (13–35%) according to the cycles of VTD and patients characteristics. But, the CR rates after 4 cycles of VTD (10.1% in VTD4 and 9.8% in VTD6) were similar with previous prospective trials. We could not exclude the possibility of underestimated CR rates because of retrospective limitations. Besides these reasons, higher gap between pre- and post-transplant CR rates can be also explained by high-quality VGPR responder.

Before the advance of proteasome inhibitors and IMIDs, a deeper response, especially post-transplant CR is a surrogate for survival [19, 20]. A novel agent-based induction therapy followed by ASCT provides further improvement in the quality of response in correlation with prolonged PFS [9]. Recently, a pooled analysis of PETHEMA/GEM clinical trials demonstrated that CR in the absence of minimal residual disease (MRD) negativity did not improve the PFS or OS compared with near-CR or PR [21]. Although the addition of MRD status to the CR assessment can accurately predict the long-term outcomes, there is no consensus about the optimal timing and methods for the assessment of MRD status, in addition to the clinical limitations in the assessment of MRD status such as, a small number of patients and the short-term follow-up duration [22]. Although our retrospective study did not assess the MRD status, the pre-ASCT CR still represented an early index of long-term survival in MM. In the multivariate analysis, the post-ASCT CR compared with PR or VGPR did not seem to be a critical prognostic relevance for PFS. It could be explained by the higher proportion of MRD-positive CR, non-sustained CR, or indolent PR similar to monoclonal gammopathy of undetermined significance [9, 23, 24]. Moreover, the post-ASCT CR compared with PR had lost prognostic significance for OS because of early intervention of next line therapy.

Bortezomib has frequently been associated with overcoming adverse prognoses in the treatment of cytogenetically high-risk MM [25]. In this study, a prolonged PFS after two additional cycles of VTD was observed in patients with R-ISS stage I/II (HR = 0.36, *P* = 0.039). Two more cycles of VTD in addition to VTD4 induction therapy might not give the extra benefit to the patients with a poor prognosis of R-ISS stage III. This result, consistent with previous studies, which showed that the attainment of CR had no significant impact on the outcome of patients with high-risk cytogenetics, long-term survival was only shown in the case of MRD negativity [21]. For patients with high-risk MM, more intensive induction therapy that overcomes poor prognosis should be administered for a high quality of response.

The incidence of PN required dose reduction was similar; however, as the intensity of dose reduction increased with exposure in the VTD4 group, patients with better tolerability were included in the VTD6 group. Previous studies on cumulative and dose-related PN reported incidence rates of 14% for grades 3 and 4 PN with 6 cycles of VTD and 10% with 3 cycles [11, 12, 26] . Unfortunately, in real practice, this study observed that almost 34.4% for the patients in cycle 5 and 6 experienced PN.

In conclusion, our results demonstrated that two additional VTD induction therapy cycles increased the pre-ASCT CR rate for NDMM. However, the PFS benefit was observed only in patients with R-ISS stage I/II disease. As a part of the effort to identify subgroups of patients who will receive the greatest benefit from two additional VTD cycles, we can recommend the patients who achieved PR only after four cycles of VTD and were tolerable to PN, excluding patients with R-ISS stage III. For patients with high-risk MM, intensive induction therapy to overcome poor prognostic factors should be administered to improve long-term outcomes.

## Supplementary information

Table S1. The probability of achieving higher quality of response by Revised International Staging System

Table S2. Factors affecting on progression-free survival for patients who remained partial response after 4 cycles of VTD induction therapy

Table S3. Factors affecting on progression free survival for patients with stage I/II by Revised-International Staging System

supplementary figures

## References

[1] Ocio EM, Richardson PG, Rajkumar SV, Palumbo A, Mateos MV, Orlowski R (2014). New drugs and novel mechanisms of action in multiple myeloma in 2013: A report from the International Myeloma Working Group (IMWG). Leukemia.

[2] Moreau P, Attal M, Pégourié B, Planche L, Hulin C, Facon T (2011). Achievement of VGPR to induction therapy is an important prognostic factor for longer PFS in the IFM 2005-01 trial. Blood.

[3] Kim JS, Kim K, Cheong JW, Min YH, Suh C, Kim H (2009). Complete remission status before autologous stem cell transplantation is an important prognostic factor in patients with multiple myeloma undergoing upfront single autologous transplantation. Biol Blood Marrow Transpl.

[4] Wang M, Delasalle K, Feng L, Thomas S, Giralt S, Qazilbash M (2010). CR represents an early index of potential long survival in multiple myeloma. Bone Marrow Transpl.

[5] Moreau P, San Miguel J, Sonneveld P, Mateos MV, Zamagni E, Avet-Loiseau H (2017). Multiple myeloma: ESMO Clinical Practice Guidelines for diagnosis, treatment and follow-up. Ann Oncol.

[6] Stewart AK, Richardson PG, San-miguel JF (2009). How I treat multiple myeloma in younger patients. Blood.

[7] Moreau P, Avet-Loiseau H, Harousseau JL, Attal M (2011). Current trends in autologous stem-cell transplantation for myeloma in the era of novel therapies. J Clin Oncol.

[8] Palumbo A, Anderson K (2011). Multiple myeloma. N Engl J Med.

[9] Cavo M, Rajkumar SV, Palumbo A, Moreau P, Orlowski R, Bladé J (2011). International Myeloma Working Group consensus approach to the treatment of multiple myeloma patients who are candidates for autologous stem cell transplantation. Blood.

[10] Moreau P, Avet-Loiseau H, Facon T, Attal M, Tiab M, Hulin C (2011). Bortezomib plus dexamethasone versus reduced-dose bortezomib, thalidomide plus dexamethasone as induction trthalidomide plus dexamethasone as induction treatment before autologous stem cell transplantation in newly diagnosed multiple myeloma. Blood.

[11] Cavo M, Tacchetti P, Patriarca F, Petrucci MT, Pantani L, Galli M (2010). Bortezomib with thalidomide plus dexamethasone compared with thalidomide plus dexamethasone as induction therapy before, and consolidation therapy after, double autologous stem-cell transplantation in newly diagnosed multiple myeloma: a randomised phase 3. Lancet.

[12] Rosiñol L, Oriol A, Teruel AI, Hernández D, López-Jiménez J, de la Rubia J (2012). Superiority of bortezomib, thalidomide, and dexamethasone (VTD) as induction pretransplantation therapy in multiple myeloma: a randomized phase 3 PETHEMA/GEM study on behalf of the Programa para el Estudio y la Terapé utica de las Hemopatías Malignas/Grup. Blood.

[13] Moreau P, Hulin C, Macro M, Caillot D, Chaleteix C, Roussel M (2016). VTD is superior to VCD prior to intensive therapy in multiple myeloma: Results of the prospective IFM2013-04 trial. Blood.

[14] Rajkumar SV, Dimopoulos MA, Palumbo A, Blade J, Merlini G, Mateos MV (2014). International Myeloma Working Group updated criteria for the diagnosis of multiple myeloma. Lancet Oncol.

[15] Palumbo A, Avet-Loiseau H, Oliva S, Lokhorst HM, Goldschmidt H, Rosiñol L (2015). Revised international staging system for multiple myeloma: A report from international myeloma working group. J Clin Oncol.

[16] Rajkumar SV, Harousseau JL, Durie B, Anderson KC, Dimopoulos M, Kyle R (2011). Consensus recommendations for the uniform reporting of clinical trials: report of the International Myeloma Workshop Consensus Panel 1. Blood.

[17] Durie BG, Harousseau JL, Miguel JS, Bladé J, Barlogie B, Anderson K (2006). International uniform response criteria for multiple myeloma. Leukemia.

[18] Coustan-Smith E, Mullighan CG, Onciu M, Behm FG, Raimondi SC, Pei D (2009). Early T-cell precursor leukaemia: a subtype of very high-risk acute lymphoblastic leukaemia. Lancet Oncol.

[19] Alexanian R, Weber D, Giralt S, Dimopoulos M, Delasalle K, Smith T (2001). Impact of complete remission with intensive therapy in patients with responsive multiple myeloma. Bone Marrow Transpl.

[20] Lahuerta JJ, Mateos MV, Martínez-López J, Rosiñol L, Sureda A, de la Rubia J (2008). Influence of pre- and post-transplantation responses on outcome of patients with multiple myeloma: Sequential improvement of response and achievement of complete response are associated with longer survival. J Clin Oncol.

[21] Lahuerta JJ, Paiva B, Vidriales MB, Cordón L, Cedena MT, Puig N (2017). Depth of response in multiple myeloma: A pooled analysis of three PETHEMA/GEM clinical trials. J Clin Oncol.

[22] Kumar S, Paiva B, Anderson KC, Durie B, Landgren O, Moreau P (2016). International Myeloma Working Group consensus criteria for response and minimal residual disease assessment in multiple myeloma. Lancet Oncol.

[23] Paiva B, Vídriales MB, Rosiñol L, Martínez-López J, Mateos MV, Ocio EM (2013). A multiparameter flow cytometry immunophenotypic algorithm for the identification of newly diagnosed symptomatic myeloma with an MGUS-like signature and long-term disease control. Leukemia.

[24] Barlogie B, Anaissie E, Haessler J, van Rhee F, Pineda-Roman M, Hollmig K (2008). Complete remission sustained 3 years from treatment initiation is a powerful surrogate for extended survival in multiple myeloma. Cancer.

[25] Bergsagel PL, Mateos MV, Gutierrez NC, Rajkumar SV, San Miguel JF (2013). Improving overall survival and overcoming adverse prognosis in the treatment of cytogenetically high-risk multiple myeloma. Blood.

[26] Richardson PG, Sonneveld P, Schuster MW, Edward A, Facon T, Harousseau JL (2009). Reversibility of symptomatic peripheral neuropathy with bortezomib in the phase III APEX trial in relapsed multiple myeloma: impact of a dose-modification guideline. Br J Haematol.

