# Supplementary material for: Benefits of additional cycles of bortezomib/thalidomide/dexamethasone (VTD) induction therapy compared to four cycles of VTD for newly diagnosed multiple myeloma
Source: Bone Marrow Transplant. 2019 Jul 29;54(12):2051–9. doi: 10.1038/s41409-019-0629-7 (PMC9763096; doi:10.1038/s41409-019-0629-7)
Supplement: Supplementary file 4 — supplementary figures [file 41409_2019_629_MOESM4_ESM.docx]

**Supplementary Figure 1. Response after two additional cycles of VTD.**


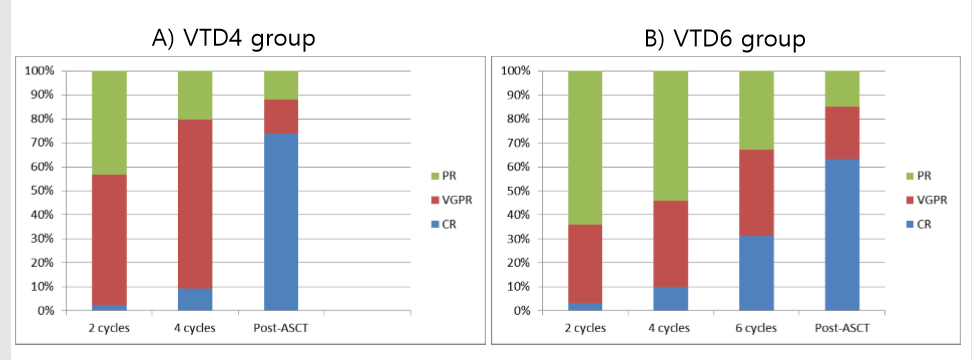


VTD4 indicates four cycles of bortezomib, thalidomide, and dexamethasone; and VTD6 indicates six cycles of bortezomib, thalidomide, and dexamethasone.

**Supplementary Figures 2. Survival rates according to the Revised International Staging System (R-ISS).**

A) The 2-year progression-free survival was 82.2% ± 6.2%, 68.0% ± 6.2%, and 47.4% ± 8.7% for stage I, II, and III R-ISS, respectively (P < 0.001). B) The 2-year overall survival was 100%, 89.6% ± 3.6%, and 82.5% ± 6.6% for stage I, II, and III R-ISS, respectively (P = 0.010).


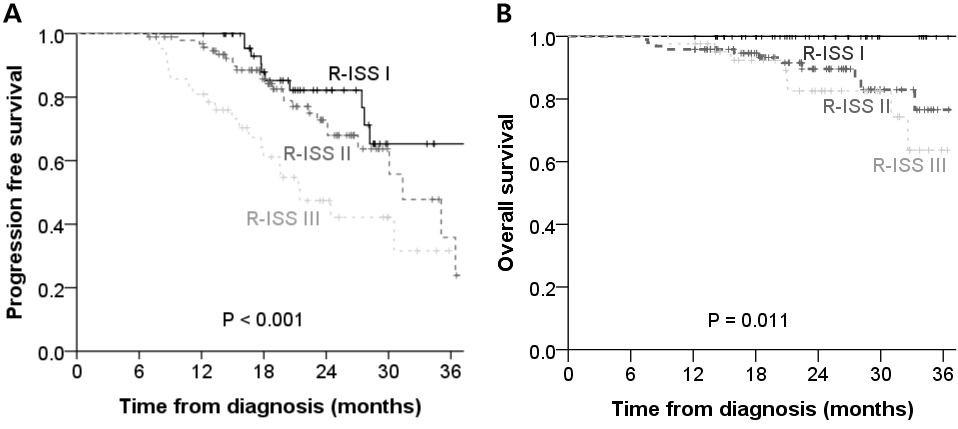


**Supplementary Figure 3. Incidence rate of adverse events that required dose reduction in the VTD4 and VTD6 groups.**


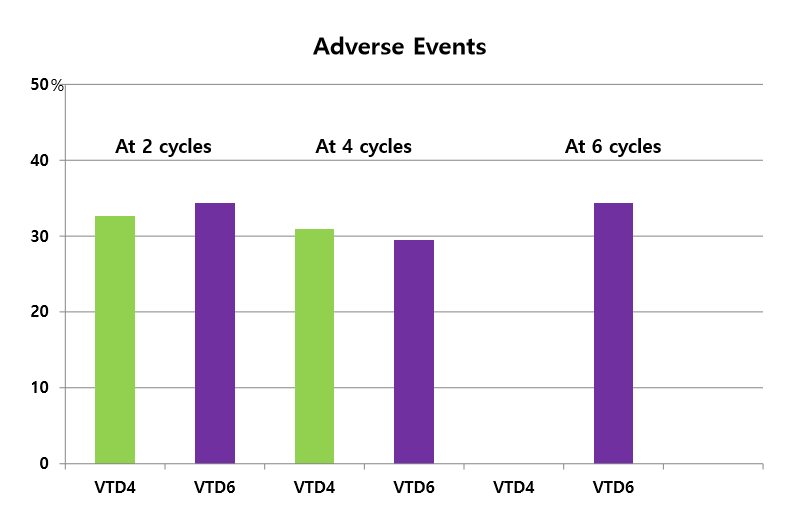


VTD4 indicates four cycles of bortezomib, thalidomide, and dexamethasone; and VTD6 indicates six cycles of bortezomib, thalidomide, and dexamethasone
